# A De Novo *CaSR* Missense Variant in Combination with Two Inherited Missense Variants in *CFTR* and *SPINK1* Detected in a Patient with Chronic Pancreatitis

**DOI:** 10.3390/biomedicines12061278

**Published:** 2024-06-09

**Authors:** Piera Bontempo, Cecilia Surace, Lucia Menale, Claudia Alicata, Gemma D’Elia, Anna Cristina Tomaiuolo, Daniele Minervino, Elisa Lorefice, Antonio Novelli

**Affiliations:** Translational Cytogenomics Research Unit, Bambino Gesù Children’s Hospital, IRCCS (Istituto di Ricovero e Cura a Carattere Scientifico), 00146 Rome, Italy; cecilia.surace@opbg.net (C.S.); luciamenale@yahoo.it (L.M.); claudia.alicata@opbg.net (C.A.); gemma.delia@opbg.net (G.D.); acristina.tomaiuolo@opbg.net (A.C.T.); daniele.minervino@opbg.net (D.M.); elisa.lorefice@opbg.net (E.L.); antonio.novelli@opbg.net (A.N.)

**Keywords:** chronic pancreatitis, next generation sequencing, trypsin pathway, *CaSR*, CFTR, SPINK1

## Abstract

Chronic pancreatitis is often secondary to alcohol abuse, but pancreatitis with no other aetiology is frequently associated with variants in genes encoding proteins related to zymogen granule activation. Our goal was to identify genomic variants in a patient by analyzing an extended panel of genes associated with the intra-pancreatic activation of the trypsin pathway. A 23-year-old woman was addressed at our institution because of chronic pancreatitis of unknown aetiology presenting recurrent episodes since she was the age of four. Next Generation Sequencing was performed to analyze a panel of nine genes associated with pancreatitis (*CaSR*, *CFTR*, *CPA1*, *CTRC*, *CTSB, KRT8*, *PRSS1*, *PRSS2*, and *SPINK1*). Three missense variants were found: p.Leu997Phe, maternally inherited, in the *CFTR* gene; p.Ile73Phe, paternally inherited, in the *SPINK1* gene; and p.Phe790Ser, a de novo variant, in the *CaSR* gene. They were classified, respectively as probably benign, a Variant of Uncertain Significance, and the last one, which has never been described in the literature, as likely being pathogenic following American College of Medical Genetics and Genomics standard guidelines. Extensive intra-pancreatic activation of trypsin pathway gene sequencing detected rare variants that were not found with other gene screening and showed that variants in different genes may interact in contributing to the onset of the pancreatitis phenotype.

## 1. Introduction

Two types of pancreatitis, sharing clinical outcomes and etiological and genetic risk factors, are currently known, namely acute and chronic pancreatitis [1]. The latter refers to a progressive inflammation of the pancreas causing abdominal pain and inducing irreversible morphological changes, which damage the organ at varying degrees and affect adjacent and distal organs.

Pancreatic acinar cells secrete digestive enzymes, in particular, they produce the pancreatic protease trypsin in an inactivated form, named trypsinogen, which is activated in the duodenum by serine enteropeptidase through the proteolytic cleavage of a hexapeptide at the N-terminus of the protein [2]. Trypsin, in turn, is able to activate chymotrypsinogens, proelastases and procarboxypeptidase B1 (CPB1) while the conversion of procarboxypeptidases A1 (CPA1) and A2 (CPA2) into their active forms requires the combined action of trypsin and chymotrypsin C (CTRC) [3]. Trypsinogen has a unique feature of auto-activation inside the pancreas, giving rise to the pancreatic pathology in certain cases [2].

The majority of the pancreatitis risk genes code for trypsin-dependent pathway proteins. These include serine protease inhibitor Kazal type 1 (SPINK1), whose function is to prevent the premature intra-pancreatic activation of trypsinogen, Serine Protease 1 and 2 (PRSS1 and PRSS2, respectively), corresponding with two isoforms of trypsinogen, Chymotripsin C (CTRC), acting as a mediator of the trypsinogen-to-trypsin conversion, Keratin 8 (KRT8), which forms a functional dimer with keratin 18 (KRT18) to maintain the structural integrity of epithelial cells, Cystic Fibrosis transmembrane conductance regulator (CFTR), a chloride channel that controls ion and water secretion and absorption in epithelial tissues, Cathepsin B (CTSB), a lysosomal cysteine protease with both endopeptidase and exopeptidase activity that plays a role in trypsinogen activation, Carboxypeptidase A1 (CPA1), which is involved in the cleavage of the C-terminal branched-chain and aromatic amino acids from dietary proteins, and the Calcium-sensing receptor, which detects increases in extracellular calcium levels (CaSR) [4,5,6,7,8,9,10,11].

The latter is a plasma membrane-bound G-protein-couples receptor expressed in the parathyroid gland, bone, intestine, kidney, brain and both acinar and duct cells of the pancreas. Here, it responds to high calcium concentrations in the juice by increasing ductal fluid secretion, thereby preventing stone formation and pancreatitis [9,12].

The *CaSR* gene (OMIM 601199) maps on 3q13.3-q21, is 120 kb long and accounts for eight exons. The cDNA encodes a predicted 120 kD polypeptide containing a large extracellular domain and seven membrane-spanning regions characteristic of G protein-coupled cell surface receptors.

The *SPINK1* gene (OMIM 167790) is located on 5q32 and is six exons long. The trypsin inhibitor encoded by this gene is secreted from pancreatic acinar cells into pancreatic juice and it prevents the trypsin-catalyzed premature activation of zymogens within the pan-creas and the pancreatic duct.

The *CFTR* gene (OMIM 602421) maps on 7q31.2 and consists of 27 exons. The encoded protein is a chloride-channel-controlling ion and performs water secretion and absorption in epithelial tissues. Pathogenic variants in this gene cause cystic fibrosis, the most common genetic disorder in European populations. Moreover, it recovers an important role in pancreas functionality, clearly increasing the risk of developing pancreatitis. It was previously known that genetic variants in *CFTR* with a loss-of-function can induce pancreatitis or exacerbate existing inflammation.

Here, we present a female patient affected by chronic pancreatitis carrying inherited variants in both *SPINK1* and *CFTR* genes and a de novo, likely pathogenic variant in the *CaSR* gene.

## 2. Detailed Case Description

### 2.1. Clinical History

The patient was a twenty-three-year-old Italian female with clinical manifestations characterized by episodes of pancreatitis with several hospitalizations. The patient was born at term to non-consanguineous, healthy Italian parents, with a healthy older brother and no family history of pancreatitis or genetic disease. The course of the pregnancy and delivery was uneventful.

At the age of six, she was diagnosed with chronic pancreatitis; in 2015, she underwent a pancreatico-jejunostomy surgery with L-L anastomosis on a Roux loop. Following the operation, the patient had one year of well-being, with occasional episodes of pancreatic pain treated sporadically with tramadol but not hospitalizations.

### 2.2. DNA Extraction and NGS Analysis

Peripheral blood samples were obtained from the proband and her family members. Genomic DNA was extracted and purified using the QIAsymphony DNA Mini Kit (Qiagen, Hilden, Germany) on a QIAsymphony automatic extractor (Qiagen) in order to obtain high-molecular-weight DNA, not degraded and with 260/280 ratios of approximately 1.8 and 260/230 of approximately 2.0. DNA quality was assessed using the NanoDrop 8000 spectrophotometer (Thermo Fisher Scientific, Waltham, MA, USA). The concentration of the DNA samples was evaluated using the FLx800 Fluorescence Microplate Reader fluorimeter with the graphical support of Gen5TM 2.0 Data Analysis software (BioTek, Winooski, VT, USA). The fluorescence signal was obtained using Quant-iT™ PicoGreen™ dsDNA Assay Kits (Molecular Probes, Eugene, OR, USA). A panel of genes associated with the intra-pancreatic activation of trypsin (IPAT) pathway has been analyzed. It includes the following genes: *SPINK1*, *PRSS1*, *PRSS2*, *CTRC*, *KRT8*, *CFTR*, *CTSB*, *CPA1* and *CaSR.* Whole exome sequencing was performed on the proband and the parents’ genomic DNA using the Twist Human Core Exome Kit (Twist Bioscience) for IPAT genes according to the manufacturer’s protocol, and sequenced on the Illumina NovaSeq6000 platform (Illumina, San Diego, CA, USA). The results were confirmed using standard Sanger exon sequencing on the proband and her parents DNA samples re-extracted from peripheral blood. The Geneyx platform (https://geneyx.com/ (accessed on 7 February 2021)) was employed for prioritization, filtering and the interpretation of the genomic data; hg37/hg19 was used as reference genome. Sequencing data were analyzed using Integrative Genomics Viewer IGV_2.10.0. The pathogenicity of the filtered candidate variants was determined according to the ACMG (American College of Medical Genetics and Genomics) guidelines and was described in standard terms.

### 2.3. Protein Analysis of CaSR

The online tool Varsome (https://varsome.com (accessed on 6 July 2021)) was used to predict the effect of genetic variants on the function of CaSR. Furthermore, 3D molecular modeling analysis was performed to show the variations in protein structure. The human CaSR model was downloaded in the AlphaFold dataset [13], and the effect of variants on this 3D model was predicted by AlphaFold [14]. The pathogenicity of the new variant was assessed using both the prediction software AlphaMissense v.4 (updated on 6 May 2024, UniProt ID P41180) and Varsome, the latter with the following parameters: PM2 moderate and PB1 supporting.

### 2.4. Sequencing and Pathogenicity Analysis

Genetic analysis revealed how the proband carries three heterozygous missense variants in three different genes of the IPAT pathway, namely *CTFR*, *SPINK1* and *CaSR*.

The c.2991G > C (p.Leu997Phe) variant in *CFTR* localizes in exon 19 and arises from a G to a C substitution, is maternally inherited and, according to the literature, is re-classified as probably pathogenetic [15]. The CFTR2 database (http://www.cftr2.org (accessed on 2 May 2021)) classifies L997F as a non-CF-causing variant. This statement is based on the evaluation of the clinical characteristics of patients carrying this variant, the functional testing of this variant, and finding this variant (combined with a CF-causing variant) in individuals who do not have CF. Although this variant has been known since 1992, its pathogenic role is still unclear. What is known is that in subjects with idiopathic pancreatitis, the p.Leu997Phe variant has also been associated with variants in the *SPINK1* gene, such as p.Asn34Ser [16].

**Figure 1:**
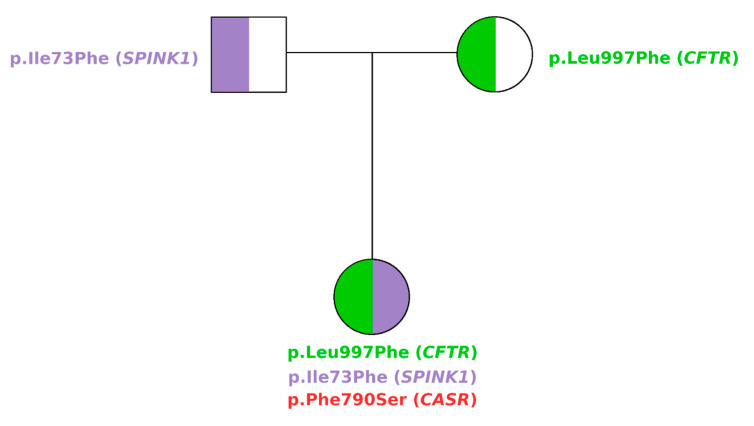
The pedigree of the family.

**Figure 2:**
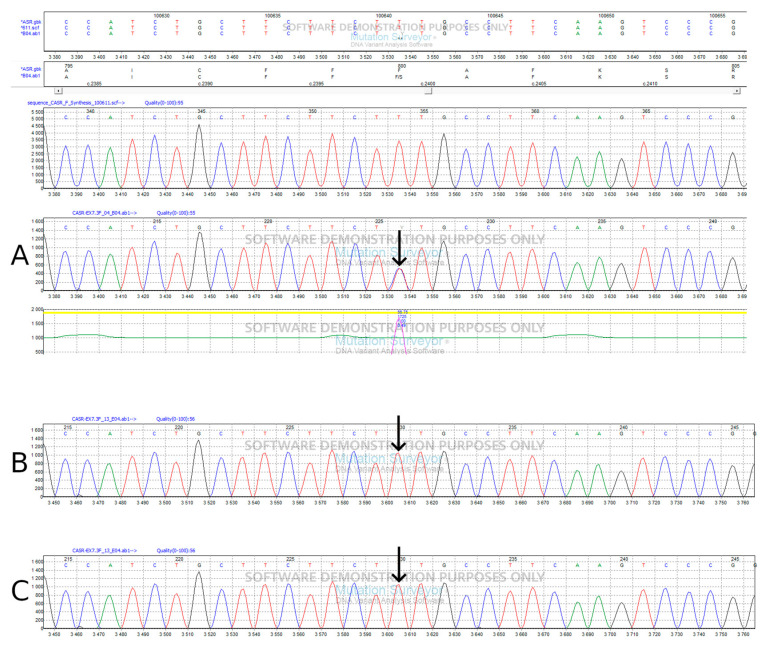

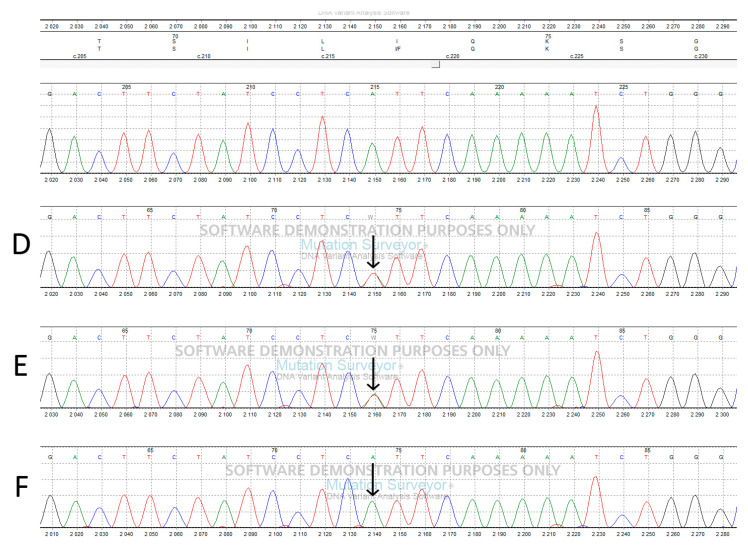
The Sanger sequencing of *CASR* exon 7 revealing c.2379T > C (p.Phe790Ser) in the proband (**A**), but not in her father (**B**) and mother (**C**). The Sanger sequencing of *SPINK1* exon 4 revealing c.217A > T (p.Ile73Phe) in the proband (**D**) and in her father (**E**), but not in her mother (**F**).

**Figure 3:**
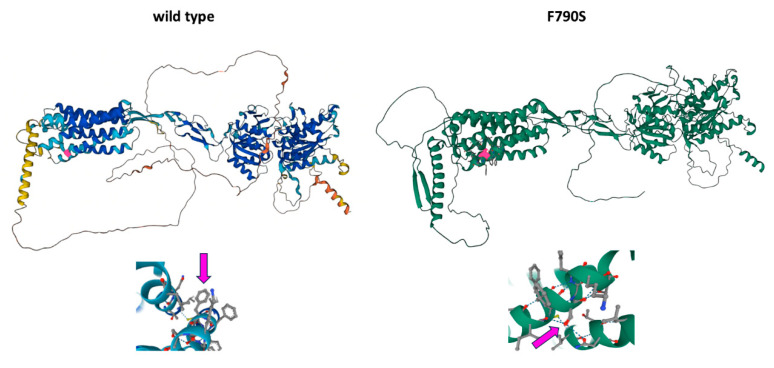
The upper left panel shows the 3D model of the wild-type CaSR protein, and on the upper right is the Phe790Ser variant. Bottom panels represent a zoom-in of the tertiary structure surrounding position 790. Pink blocks on top and pink arrows on the bottom indicate the residue at position 790.

The c.217A > T variant (p.Ile73Phe), inherited from the father, maps on coding exon 4 of the *SPINK1*. The isoleucine at codon 73, which is conserved over species, is replaced by phenylalanine, an amino acid with highly similar properties. So far, c.217A > T has not been reported in individuals with chronic pancreatitis nor has its impact on protein function been evidenced. In our knowledge, this is the first report where p.Ile73Phe is described and, based on the evidence outlined above, the variant was classified as having uncertain significance (VUS). The c.2379T > C (p.Phe790Ser) variant of *CaSR* occurred de novo (Figure 1 and Figure 2), in the absence of the patient’s family history of Familial Hypocalciuric Hypercalcemia, has not been previously reported and, here, we provide the first report on this variant. Both the Varsome [17] and AlphaMissense variant effect prediction software categorized this variant as likely to be deleterious; in particular, AlphaMissense assigns it a 0.781 pathogenicity score. We classified variants as pathogenic if the score exceeded 0.564, and classified variants as benign if the score was below 0.34, as recommended by the authors [18]. The 3D model predicted that the variant shows similar features to the wild-type (wt) protein (Figure 3) despite the fact that the amino acid present in the p.Phe790Ser variant has different chemical properties compared to the wt.

## 3. Discussion

In this manuscript, we report a genetic study performed on a female patient with pancreatitis. By combining molecular genetic testing and an in silico prediction of pathogenicity, we found that the proband carries two missense variants in *SPINK1* and *CFTR* inherited from her father and her mother, respectively, in combination with a missense variant in *CaSR* that occurred de novo.

A group of *CFTR* variants has been found to be in statistically significant association with chronic pancreatitis. In particular, p.Arg117His and p.Leu967Ser were significantly overrepresented in cases relative to controls, whereas p.Arg74Gln, p.Arg75Gln, p.Arg170His, p.Asp1152His, p.Ser1235Arg, p.Asp1270Asn and p.Leu997Phe were not found to be in statistically significant association with chronic pancreatitis when analyzed individually, due to their relatively rare occurrence. However, although their pooled analysis indicated a modest association, Berke and colleagues state that the effect of the individual variants cannot be determined with confidence until more data becomes available [19]. Indeed, despite the fact that the homozygosity for p.Leu997Phe does not give rise to any clinical symptoms, if it is present in compound heterozygosity with another known pathogenic variant, it may contribute to the appearance of CFTR-related pathologies (CFTR2 database: http://www.cftr2.org (accessed on 2 May 2021)). We therefore surmise that whilst the mutation of p.Leu997Phe may be insufficient to cause disease on its own, it may well act synergistically with other genetic factors to confer an increased risk of cystic fibrosis-related disease and pancreatitis.

The co-inheritance of *SPINK1* and *CFTR* variants has been identified in patients with early onset chronic pancreatitis or recurrent acute pancreatitis [20]. Genetic studies on pancreatitis patients identified that the p.Arg75Gln variant of *CFTR* in combination with p.Asn34Ser and p.Pro55Ser variants of *SPINK1* causes a selective defect in bicarbonate conductance and increases the risk of pancreatitis [21].

Similar to *CFTR* mutations, the exact contribution of *CaSR* variants to pancreatitis risk remains controversial, although it has been demonstrated that these variants may impair calcium sensing and promote higher ductal Ca^2+^ levels [22]. Recent studies in the literature reported that common *CaSR* variants do not alter the risk of CP and should not be considered as responsible for genetic risk in subjects suffering from pancreatitis [23,24]. Instead, our patient shows p.Phe790Ser, a de novo *CaSR* variant, classified as probably pathogenic, and its interaction with the other two described variants, p.Leu997Phe (*CFTR*) and p.Ile73Phe (*SPINK1*), is strongly suggestive of the pancreatic phenotype. Heterozygous inactivating variants in *CaSR* cause Familial Hypocalciuric Hypercalcemia (FHH), an autosomal dominant disorder characterized by elevated serum calcium and decreased urinary calcium excretion [25,26]. In a small number of FHH patients, trans-heterozygosity for *SPINK1* and *CaSR* variants has been documented [27,28]. *CaSR* variants seem not to have a direct local effect in the pancreas; indeed, FHH-associated pancreatitis is likely to be due to hypercalcemia, which is a well-known risk factor for pancreatitis [29].

## 4. Conclusions

Based on these observations, it is unlikely that the de novo variant in the *CaSR* gene present in our patient is causative of the phenotype in a stand-alone manner. Importantly, our patient inherited a *CFTR* variant from her healthy mother and a *SPINK1* variant from her healthy father. Taken together, these data suggest that the combination of the three variants may contribute to the onset of the pancreatitis in the patient.

In conclusion, the analysis of IPAT pathway genes plays an important role in the identification of genetic risk factors of pancreatitis. This has major relevance in pediatric patients, for whom an early diagnosis can address specific medical approaches, with positive repercussions on the health and the quality of life of the patients. The involvement of the three described genes (*CaSR*, *CFTR*, and *SPINK1*) in the same pathway, never reported together before, supports their causative role in our patient by means of a trans-heterozigosity mechanism. Further studies are needed to disclose their interactions and functional studies will clarify how they act, especially the mutated CaSR protein.

## Data Availability

The data presented in this study are available on request from the corresponding author. The data are not publicly available due to privacy issues (single case report).
